# Relevance of Neonatal Behavior Assessment Scale for Infants With Somatic Disorders: Comparison on One Matched Group of Control

**DOI:** 10.3389/fped.2020.506384

**Published:** 2021-01-13

**Authors:** Rose-Angélique Belot, Margaux Bouteloup, André Mariage, Drina Candilis-huisman, Nicolas Mottet, Denis Mellier

**Affiliations:** ^1^Laboratory of Psychology EA3188, UFR SLHS, University of Bourgogne/Franche-Comte, Besançon, France; ^2^Research Center “Psychanalyse et Médecine” EA 3522, Paris Diderot University, Paris, France; ^3^Department of Obstetrics and Gynecology, Besançon Regional University Hospital, Besançon, France

**Keywords:** infants 5–18 weeks, somatic disorders, emotion, regulation, care, NBAS

## Abstract

**Objective:** To compare the Neonatal Behavior Assessment Scale results in two groups of infants with or without somatic disorder (*N* = 26).

**Method:** The Neonatal Behavior Assessment Scale was administered to two groups (clinical and control) of 13 infants each, aged from 5 to 18 weeks, matched 2 by 2 according to sex, age, rank among siblings, and parental socio-professional category. The first group includes infants with somatic disorder (clinical) and is matched with a second group of “healthy infants” (control).

**Results:** Results indicate that the mean score of the control group is significantly higher than that of the clinical group. Most of the items are affected by the presence of a somatic disorder. Indeed, five out of the six categories present a statistically significant difference in favor of the control group, more specifically for the items “state regulation,” “motor system,” and “orientation/interaction.”

**Conclusion:** This exploratory research enables a precise description of infants' difficulty in regulating excitations and the impact of somatic disorders on their development. This innovative knowledge will assist pediatricians and health professionals in the understanding of infants' characteristics to develop an adapted-care.

## Introduction

Early somatic disorders in the baby are complex because many parameters must be taken into account. Eating and sleeping are central functions that are particularly important during the first year (1) because they require autonomous internal regulation to be established (hunger, satiety, and circadian rhythm). Twenty-five percent of infants developing normally present eating disorders (2, 3) with no link to any growth disorder. In addition, a literature review evidenced the reported presence of colic in 5–40% of infants (4) and painful gastroesophageal reflux in 5–30% of infants in the first weeks of life. Colic predominates among somatic disorders in infants, accounting for more than a third (5).

Sleeping disorders are difficult to characterize and are regularly underestimated because there is no circadian rhythm during the first months of an infant's life. Indeed, before 2–3 months of age, infants tend to follow an ultradian pattern in which four stages alternate: quiet wakefulness, agitated wakefulness, quiet sleep, and agitated sleep (6).

The prevalence of sleeping disorders is known to be 30% (7) in under 2-year-olds, with 75% recognized as behavioral disorders (8). Furthermore, idiopathic colic in infants is a characteristic somatic sign contributing to other bodily manifestations in the first year of life (9). Five percent of infants present breath-holding spells, a proportion that is equivalent in girls and boys.

Infants resort very early on to rudimentary self-regulation strategies, such as the neurophysiological mechanisms of sucking and other movements. These mechanisms become more complex with age (10, 11). Because the infant's nervous system is immature, it cannot on its own manage a high level of excitation. The mother's role as a protective shield to her child's excitation is primordial (12, 13) until the formation of the infant's psychic envelopes (14, 15).

Many studies have shown that difficulties in emotional regulation are linked to eating disorders (16, 17).

In infants with psychosomatic disorders, emotional regulation is weak and inadequate in the early months. The infant is overwhelmed by internal and external excitations.

His entourage is powerless to help him calm down (18–20).

Observation and the application of the Neonatal Behavioral Assessment Scale (NBAS) (21–23) provide a privileged and relevant tool to thoroughly assess an infant's development and abilities and emotional regulation: signs of stress, motor and tonic abilities, excitability, irritability, appeasement and self-appeasement, alertness, orientation, and relational skills. The NBAS evidences an infant's contribution and his abilities to model his interactions: sensory characteristics (sight, sense of smell, and hearing), motor characteristics (muscular tension and diverted attention), and kinesthetic characteristics.

The NBAS is used in many research areas. This scale is an excellent tool to appreciate the infant's development and behavior and the risk factors associated such as prematurity (24–26), the influence of life *in utero* in certain cultural contexts (27), a wide variety of cultural contexts (28–31), and the effects of post-natal depression on infant (32, 33). Other studies using the NBAS methodology focus on the neonatal behavior of infants at familial risk for attention deficit hyperactivity disorder (34). The NBAS scores also permit to predict the infant's development: these scores accounted for 21–47% of the variance in developmental outcome at 36 months of age (35).

Only two studies reported using the NBAS for infants with colic (36, 37). Covington demonstrated that the scores of infants with colic were lower for orientation items. Keefe shows that two components of the NBAS were related to the development of colic or infant irritability at 1 month of age.

Another study shows that results at the NBAS are related to cerebral blood flow velocity asymmetry for premature infants (38). There is, therefore, an interest to explore by using NBAS the competencies and aptitudes of infants presenting somatic disorders.

## Methods

This research originates from a thesis: all ethical principles have been taken into account, and all written informed consents have been obtained. At the moment of the study, under French legation, ethics committee approval was not requested in the case of this non-interventional study.

### Sample

The study focused on comparing two groups (clinical and control) of 13 infants each, aged ~5–18 weeks and their families, matched 2 by 2 according to sex, age, rank among siblings, and parental socio-professional category (see Tables 1, 2). The infants in the clinical group were seen in the hospital, and the infants in the control group were seen in nurseries.

**Table 1:** Characteristics of the control and clinical groups.

**Clinical group**	**Control group**
•with somatic disorders •*n* = 13 •Age: 5–18 weeks •constituted by pediatricians in hospital	•without somatic disorders •*n* = 13 •matched and according to sex, age, rank among siblings, and parental socio-professional categories •constituted by manager in day-nursery

*Comparison of two groups. (Institutional approvals and individual consents obtained)*.

**Table 2:** Population: children matched for sex, age, socio-professional category, and rank among siblings.

**Family and allocated group**	**Sex**	**First name/anonymity**	**Age M month D Days**	**Nature of somatic expression**	**Family make-up and siblings**	**Prof. father**	**Age father, years**	**Prof. mother**	**Age mother, years**
**Mr. and Mrs A. clinical**	Boy	Alexis	3M 22D	3rd episode bronchiolitis	2nd child of couple and mother (1G, 5 years)	Cat. 5	39	Cat. 4	30
Mr. and Mrs N. control	B	Ulysse	3M 22D	*No somatic expression*	*2nd child of mother*, 1 B, 18 years 1st child of couple	Cat. 5	33	Cat. 4	37
**Mr. and Mrs B. clinical**	B	Tristan	1M 28D	GERD—Crying, agitation, sleep disorders	*1st child of mother and couple* (Father has 3 adolescent G)	Cat. 3	46	Cat. 3	31
Mr. and Mrs O. control	B	Désiré	2M 3D	*No somatic expression*	1st child of couple	Cat. 3	39	Cat. 3	36
**Mr. and Mrs C. clinical**	Girl	Inès	1M 19D	Spells of sobs	1st child of couple	Cat. 6	28	Cat. 6	29
Mr. and Mrs P. control	G	Mélodie	1M 11D	*No somatic expression*	1st child of couple	Cat. 6	35	Cat. 6	27
**Mr. and Mrs D. clinical**	G	Blanche	2M 7D	Food refusal	3rd child of couple (2 G, 4 and 7 years)	Cat. 5	33	Cat. 8	32
Mr. and Mrs Q. control	G	Diane	2M 7D	*No somatic expression*	3rd child of couple	Cat. 5	39	Cat. 8	27
**Mr. and Mrs E. clinical**	B	Pierre	3M 8D	3rd episode bronchiolitis	3rd child of couple (2 G, 3 and 5 years)	Cat. 4	36	Cat. 4	29
Mr. and Mrs R. control	B	Remy	3M	*No somatic expression*	3^è*me*^ enfant du couple (1 G, 3 years and 1 B, 7 years)	Cat. 4	34	Cat. 4	33
**Mr. and Mrs F. clinical**	B	Virgile	1M 13D	Colic. Sleep disorders. Agitation	1st child of couple	Cat. 6	24	Cat. 6	28
Mr. and Mrs S. control	B	Elie	1M 13D	*No somatic expression*	1st child of couple	Cat. 6	23	Cat. 6	24
**Mr. and Mrs G. clinical**	G	Alma	2M	Spells of sobs—crying	1st child of couple–*2nd child of mother*, 1 B, 19 years	Cat. 4	33	Cat. 3	37
Mr. and Mrs T. control	G	Anna	2M	*No somatic expression*	2nd child of couple (1 B 3 years)	Cat. 3	38	Cat. 4	34
**Mr. and Mrs H. clinical**	B	Gratien	3M 25D	Spells of sobs and slight oesophagitis	1st child of couple–*2nd child of mother* (1 B 3 years) 2^nd^ child of father (1 G 4 years)	Cat. 5	30	Cat. 4	24
Mr. and Mrs U. control	B	Félicien	3M 11D	*No somatic expression*	2nd child of couple (1 G 4 years)	Cat. 4	34	Cat. 4	36
**Mr. and Mrs I. clinical**	G	Eva	4M 15D	GERD	1st child of couple	Cat. 3	30	Cat. 3	30
Mr. and Mrs V. control	G	Emma	4M 12D	*No somatic expression*	1st child of couple	Cat. 3	31	Cat. 3	31
**Mr. and Mrs J. clinical**	B	Noël	3M 2D	Colic—GERD—daytime sleep disturbances	*1st child of couple and mother* (Father has 2 B, 15 and 17 years)	Cat. 6	38	Cat. 6	35
Mr. and Mrs W. control	B	Nathan	3M 2D	*No somatic expression*	1st child of couple	Cat. 6	27	Cat. 6	26
**Mr. and Mrs K. clinical**	G	Océane	1M 21D	Colic, screaming, daytime sleep disorders	*1st child of couple and mother*. Father has 2 B 16 and 17 years	Cat. 4	42	Cat. 4	37
Mr. and Mrs X. control	G	Oriane	1M 22D	*No somatic expression*	1st child of couple and mother. Father has 2 G	Cat. 4	30	Cat. 4	26
**Mr. and Mrs L. clinical**	B	Aymé	1M 8D	Daytime sleeping disorders	1st child of couple	Cat. 4	30	Cat. 4	26
Mr. and Mrs Y. control	B	Justin	1M 15D	*No somatic expression*	1st child of couple	Cat. 4	31	Cat. 4	32
**Mr. and Mrs M. clinical**	B	Madras	2M 15D	Colic—GERD—Sleep disorders—Screaming	*2nd child of mother* (1 G, 15 years) 1st child of couple and father	Cat. 5	30	Cat. 5	36
Mr. and Mrs Z. control	B	Constant	2M 12D	*No somatic expression*	*2nd child of mother* (1 B, 18 months)	Cat. 3	41	Cat. 3	36

*GERD, gastroesophageal reflux disease*.

All institutional approvals were obtained before the start of the study.

The inclusion criteria were as follows:

- full-term infants and good health aged up to 18 weeks
- somatic manifestations without organic etiology
- with no natal difficulty (no reoxygenation). All measures taken were normal.
- parents were living together and had wanted to have a child

The two groups (13 in each group) differed with respect to the absence or presence of a somatic manifestation.

The first group, referred to as the clinical group (eight boys and five girls), had somatic manifestations in the following areas: sleeping, eating, digestion, and breathing (including losses of consciousness with no organic etiology and breath-holding spells). The other 13 infants formed the control group and did not present any somatic manifestations.

The youngest infant in our study was aged 5 weeks, and the oldest was 18 weeks.

The clinical group was composed of:

- 2 children who presented gastroesophageal reflux as their main symptom−4 other children had gastroesophageal reflux, but in association with sleeping disorders, colic or fainting.
- 4 children with an association of colic and sleeping disorders,
- 2 children who had been hospitalized after a third episode of bronchiolitis,
- 1 child who had been hospitalized for refusing to be fed,
- 3 children who had been hospitalized for investigation of spells of sobs,
- 1 child had diurnal sleeping problems with no other associated somatic manifestation.

Crying and states of agitation do not constitute a specific category because they are linked to other manifestations such as colic episodes and/or sleeping disorders. In our population, several disorders could occur for the same child—for instance, apparent life-threatening event and gastroesophageal reflux. Sleeping and digestion (colic) disorders were often combined and/or occurred alongside other somatic manifestations.

Infant's somatic disorders are, by nature, varied and are quickly reversible (12, 39), mostly spontaneously or after medical and psychological care. This was the case with all the infants we met. Indeed, all the infants enjoyed a regular follow-up with a pediatrician. After the assessment using the NBAS, some families benefited from counseling.

At the age of 6 months, in our clinical population, all the infants presented a favorable evolution, conforming to the definition of punctual symptomatology.

In this context, we can consider the clinical group as homogeneous, even if there are some original diversities in somatic manifestations.

### Procedure

The pediatricians constituted the clinical group. They were informed of the inclusion criteria and offered families to participate in the research. The researcher contacted the mother to present in more detail the study and obtain an agreement and written consent. No refusal has been recorded.

The control group has been constituted by a day-nursery manager, respecting the matching criteria. The same approach for presentation and agreement was followed up. All the infants were with their mothers during the NBAS assessment.

The test was administrated by a researcher psychologist, certified to Brazelton Scale, and in the combined presence of a pediatrician for the clinical group. All the assessments took place during the somatic manifestations.

The main characteristic of the NBAS is that it records the best performance and assesses infants' ability to organize their responses in well-defined states of consciousness. The presentation of the items is, therefore, not linear but depends on the infant's state of consciousness. The behavior items are classified according to six variables to simplify data analysis: habituation, orientation–interaction, motor system, state regulation, and autonomic nervous system (ANS).

Brazelton (21, 22) and Lester (40) have recommended recoding several scale items to appreciate optimal scores homogeneously. Indeed, without recoding, the optimal scores can be 1, 5, or 9. With the recoding, number 9 corresponds to the best possible result and number 1 the lower infant's performance. In our study, it was thus necessary to recode according to Brazelton and Lester's principle.

### Statistical Analyses

Each cluster was presented with a mean and standard deviation. Comparisons (clinical group vs. control group) were performed using the Student *t*-test with SPSS version 19.0.

## Results

The means of both groups are calculated and compared for the six items of the NBAS: habituation, orientation/interaction, motor system, state organization, state regulation, and ANS (Table 3).

**Table 3:** Means for the Brazelton neonatal behavioral assessment scale and comparison of the two groups.

	**Group**			
	**Clinical**	**Control**		
	**M (SD)**	**M (SD)**	***t***	***p***
Habituation	6.60 (2.20)	8.23 (1.62)	2.06	0.05
Orientation/interaction	6.10 (2.25)	8.26 (0.94)	3.34	0.01
Motor system	4.50 (1.63)	6.46 (0.98)	3.87	0.01
State organization	2.39 (1.62)	3.80 (1.77)	2.21	0.05
State regulation	3.83 (2.36)	7.15 (1.15)	4.76	0.001
Autonomic nervous system	7.18 (1.34)	7.87 (0.29)	1.90	NS
Global mean	5.04 (1.72)	7.34 (0.47)	4.86	0.01

The only criterion that distinguished the two groups was the presence of the clinical group of a somatic manifestation: disorders relating to sleep, digestion, eating, or breathing.

First of all, the means obtained for the 26 items by the clinical group (*M* = 5.04, SD = 1.72) were significantly lower (*p* < 0.01) than those obtained by the control group (*M* = 7.34, SD = 0.47). The infants' competencies and performances were, therefore, generally greater in the control group than in the clinical group. There is one important point: to ensure that the infants in the clinical group could obtain their best possible score, the investigator had to pay considerable attention and provide support, whereas with the control group, the participation was less demanding.

Furthermore, in five out of six categories in the NBAS (habituation, orientation/interaction, motor system, state organization, and state regulation), the control group obtained significantly higher scores than those in the clinical group. Only the category ANS did not show any statistically significant difference.

The most significant differences were for the following categories: state regulation (*p* < 0.001), orientation–interaction (*p* < 0.01), and motor system (*p* < 0.01).

## Discussion

Habituation includes items such as response to light, to a rattle, and to a small bell and reflects infants' aptitude for getting used to external stimulation. The difference in scores between the control group (*M* = 8.23, SD = 1.62) and the clinical group (*M* = 6.60, SD = 2.20) was considerable and significant [t_(26)_ = 2.06, *p* < 0.05]. During the different administrations of the NBAS, we indeed observed that infants had difficulty in regulating all the endogenous and exogenous excitations that they were subjected to. For infants with somatic disorders, their sensitivity to stimuli seemed to us to be much greater than for the infants in the control group. Their habituation abilities were more difficult to mobilize. We think that this suggests a chain reaction, whereby a context of hyperactivity discourages the emergence of internal regulations and habituation skills.

Orientation–interaction includes orientation items with single or multiple visual, auditory stimuli of the inanimate type (red ball and rattle) and animate (the researcher's face and voice). The performances of the control group infants here (*M* = 8.26, SD = 0.94) were significantly better than those of the clinical group (*M* = 6.10, SD = 2.25) [t_(26)_ = 3.34, *p* < 0. 01]. Infants with somatic disorders provided weaker responses when faced with interactive and orientation stimulations.

Indeed, the various competencies explored with the NBAS—speed of habituation to stimulus, orientation and interaction with the investigator, and variety and range of the infant's motor skills—showed poor responses. Is this failure to respond encouraged by burgeoning somatic disorders, or do somatic manifestations hamper the development of these aptitudes? The question has not been addressed. It is difficult to identify the origin of the disorders, but chain reactions take effect very quickly and could amplify the initial cause, if there is one.

Our clinical observations, however, provide some insights. For instance, the children from the clinical group generally seemed less available or receptive to provide optimal reactions to the stimuli, as they were not able to stay in a quiet state of wakefulness for very long (state 4).

This means that all the other functions in the various domains are hampered. For example, their performances on orientation and interaction items are dependent on their ability to present stability in the various states and to control their level of excitation. Motor system includes the following items: general tone, motor maturity, the Moro reflex test, and the level of spontaneous and induced activity. The mean score for the control group (*M* = 6.46, SD = 0.98) was higher than for the clinical group (*M* = 4.50, SD = 1.63). This difference was also significant [t_(26)_ = 3.87, *p* < 0.01] in favor of the control group. Our observations overall showed that there was either spontaneous hyperactivity in infants with somatic disorders or the complete opposite, a lack of muscle tone and excessive slackening. For instance, in the Moro reflex test, infants with somatic disorders did not have enough muscle tone to support their heads, and their spontaneous activity was poor. Other infants with somatic disorders exhibited hypertonic performances and considerable muscular tension. The alternation of periods of tension and slackening was generally not as frequently observed in the clinical group, where tonicity was present but more flexible.

State organization includes peaks of excitation, states of increased agitation, and liability of states of wakefulness. The mean scores were also significantly different here in favor of the control group (*M* = 3.80, SD = 1.77) against (*M* = 2.39, SD = 1.62) for the clinical group. This difference is also significant [t_(26)_ = 2.21, *p* < 0.05].

The infants' responses to these items, in our view, provide a good prognosis for detecting their abilities in all the other areas studied on the NBAS. Indeed, an infant's ability to face either internal stimuli (coming from their body) or external stimuli (noise, light, and heat) constitutes major elements whereby all the infant's other aptitudes and competencies can emerge and be expressed. This involves the issue of infants' own abilities to control their excitation, the quality of the environment, and the help received in managing their excitation, both internal and external.

State regulation includes items such as cuddliness, consolability, self-appeasement, hand-to-mouth activity, and highlights infants' ability to “let themselves go” in the arms of an adult and relax in a comforting position (cuddliness item), to use their own self-consolation abilities (hand-to-mouth and self-appeasement items) and their ability to be consoled (consolability item). This is the area where infants from the clinical group obtained the lowest scores (*M* = 3.83, SD = 2.36) compared with the control group (*M* = 7.15, SD = 1.15). This difference was the most significant of the five categories studied [t_(26)_ = 4.76, *p* < 0.001].

The excessive excitation and motor agitation observed in the clinical group hindered the infants' ability to relax and hampered contact and hand-to-mouth coordination. Similarly, an infant's ability to be consoled is necessarily hindered when he is subjected to excess excitation that cannot be controlled by the infant himself nor by his environment. We noticed that the infants' self-appeasement abilities could not develop when they did not succeed in settling down to a sufficiently long state of quietude. Alongside, insufficient excitation (particularly from the environment) generated the same kind of difficulty. Infants cannot on their own regulate states of excitation that result from their main functions. Their physical and psychological immaturity requires appropriate care. In some cases, these infants present an excess of excitation that is not triggered by the environment but is residual, leading to the same consequences as described when excess excitation dominates.

The ANS includes tremors, starts, and rapid changes in skin color. The scores for the two groups were different (mean = 7.87 for the control group and mean = 7.18 for the clinical group), but the difference was not significant. This study did not consider premature infants. However, tremors and starts were more frequent in infants with somatic disorders. The stability of the ANS (as illustrated on the NBAS by the presence or absence of tremors and starts or by changes in skin color) is less affected than other domains by the presence of somatic manifestations. Indeed, infants over 1-month-old can maintain regulations inside their nervous system quite well. However, the most sensitive item in this group of items, which shows the greatest interpopulation variation, is skin color. Infants presenting a somatic manifestation are less likely to show the ability to regulate their body temperature.

Als (24), who carried out repeat assessments on 10-day-old infants, evidenced a hierarchy in the control of physiological functions and the different dimensions of behavior. According to these authors, regulation of the ANS in infants could precede motor organization, which could be followed by the potential to regulate their states. Orientation–interaction abilities could come last.

From this observation, a hierarchy within the nervous system internal regulations could be defined: blood supply could be the most sensitive regulation and, therefore, the last step in the formation of the ANS regulations.

## Conclusion

This exploratory research on 26 infants is relevant because it shows a link between the infant somatic manifestation and NBAS skills. The inclusion of a control group in this research helped shed light, via an interpopulation comparison, on the circumstances favoring satisfactory psychosomatic development in infants.

The use of the NBAS in this context, even if the sample is small, enables us to grasp the range of competences and the specific defense modes that are characteristic of the main functions: the development of sensory qualities (sight, smell, and hearing), motor qualities (muscular tension and diverted attention), and coenesthetic abilities.

This study has thus provided precise information on the differences between infants with somatic disorders and those without and in what areas these differences occur. In addition, it has enabled the identification of the strengths and weaknesses in each group, thanks to the NBAS indicators. The NBAS is a rich and fruitful investigation tool to understand what is usually known as temperament data or infants' personal initial characteristics: their modes of organization, their development dynamics, and their range of responses to disruptions and also to environmental, affective, and/or relational stimulations. The in-depth understanding of these characteristics will be helpful for pediatricians and health professionals to propose care adapted to each baby.

## Limits and Perspective of Our Study

- The promising results of this exploratory research confirm the need for in-depth research with a larger sample.
- The next NBAS assessments will be videotaped for double-blind quotes, always with certified professionals.
- A question remains: is emotional regulation impacted by somatic manifestations, or are difficulties in emotional regulation caused early symptomatology? This study cannot say clearly either way. A longitudinal study with a larger sample is required to throw more light on the issue.

## Data Availability Statement

The datasets generated for this study are available on request to the corresponding author.

## Ethics Statement

Ethical approval was not provided for this study on human participants because at the moment of the study, under French legatory, registered number and Clinical Trial registration was not requested in case of non-interventional study. This research originates from a thesis: all approvals are obtained and all ethical principles have been taken into account. Written informed consent to participate in this study was provided by the participants' legal guardian/next of kin.

## Author Contributions

R-AB devised the project, the main conceptual ideas, and proof outline. AM realized the statistical analyses. DC and NM reviewed the manuscript and provided their thoughtful comments and suggestions. R-AB, MB, and DM wrote the manuscript. All authors contributed to the article and approved the submitted version.

## Conflict of Interest

The authors declare that the research was conducted in the absence of any commercial or financial relationships that could be construed as a potential conflict of interest.
